# Supplementary figures and images for: Exploiting collateral sensitivity controls growth of mixed culture of sensitive and resistant cells and decreases selection for resistant cells in a cell line model
Source: Cancer Cell Int. 2020 Jun 17;20:253. doi: 10.1186/s12935-020-01337-1 (PMC7301982; doi:10.1186/s12935-020-01337-1)

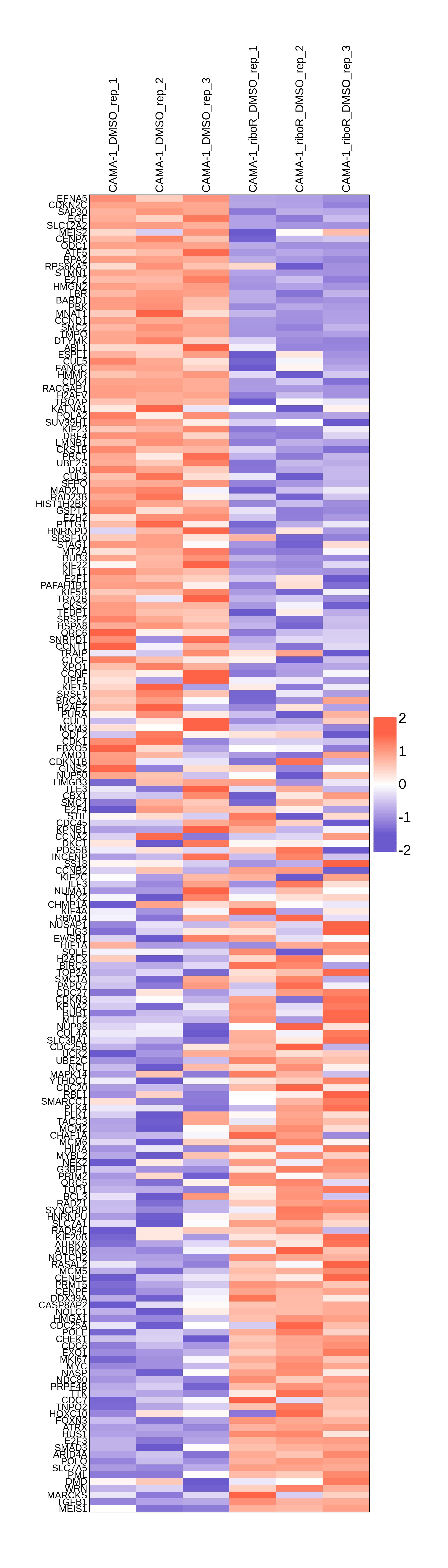

Supplement: Supplementary file 3 — Additional file 3: Figure S2. Extended heatmap of Fig. 3, Panel B incorporating gene symbols. [file 12935_2020_1337_MOESM3_ESM.png]
